# The Impact of Austerity on Mental Health Service Provision: A UK Perspective

**DOI:** 10.3390/ijerph15061145

**Published:** 2018-06-01

**Authors:** Ian Cummins

**Affiliations:** School of Health and Society, University of Salford, Salford M5 4WT, UK; i.d.cummins@salford.ac.uk

**Keywords:** austerity, mental health services, welfare reform

## Abstract

This is a discussion paper which examines the impact of austerity policies on the provision of mental health services in the United Kingdom. Austerity is a shorthand for a series of policies introduced by the Conservative and Liberal Democrat Coalition government in the UK from 2010 onwards. In response to the fiscal crisis following the bail out of the banks in 2008, it was argued that significant reductions in public spending were required. The background to these policies is examined before a consideration of their impact on mental health services. These policies had a disproportionate impact on people living in poverty. People with health problems including mental problems are overrepresented in this group. At the same time, welfare and community services are under increasing financial pressures having to respond to increased demand within a context of reduced budgets. There is increasing recognition of the role that social factors and adverse childhood experiences have in the development and trajectory of mental health problems. Mental health social workers, alongside other professionals, seek to explain mental distress by the use of some variant of a biopsychosocial model. The extent of mental health problems as a one of their measures of the impact of inequality. More unequal societies create greater levels of distress. There is a social gradient in the extent of mental health problems—the impact of severe mental illness means that many individuals are unable to work or, if they can return to work, they find it difficult to gain employment because of discrimination. The paper concludes that austerity and associated policies have combined to increase the overall burden of mental distress and marginalisation within the UK.

## 1. Introduction

Two important works—Scull’s (2015) [1] Madness in Civilisation: A Cultural History of Insanity and Foot’s (2015) [2] The Man who Closed the Asylums—highlight the barriers that had to be overcome in the struggle to humanise mental health provision. Scull’s magisterial study of societal responses to madness demonstrates that there appear to be few, if any, indignities that societies have not been prepared to inflict on citizens in the name of treatment. Foot’s study of Franco Basaglia makes very clear the essential political nature of the structure and delivery of mental health care. There is an implicit danger that, in examining the current crisis in community mental health services, the failures and abuses of the past are assigned to history. The assumption being that such progress has been made that these issues are now resolved. Macintyre et al. (2018) [3] emphasised the importance of a consideration of the links between social economic factors and the occurrence and experience of mental health problems. There have been significant moves toward this but the continued dominance of medical and individualised approaches to mental distress prevents a full consideration of the impact of socio-economic factors (Shim et al. 2014) [4]. Macintyre et al. (2018) [3] concluded that such an analysis that starts from a socioeconomic position can be the basis for a move towards a social justice approach for mental health. This paper explores these debates within the context of the impact of the social and economic policies of austerity that have been pursued in the UK since 2010.

It is now widely accepted that one needs to examine a range of social, economic, political and cultural factors when examining mental health outcomes (Murray 2017) [5].

The Marmot Review (2010) [6] identifies a clear link among poverty, inequality and poor mental health. The issues of causality are complex. However, the overall conclusion is inescapable. For example, Wilkinson and Pickett (2009) [7] used the prevalence of mental illness as one of their measures of the impact of inequality. Mental health and mental illness can thus be regarded as signifiers of the broader nature of society. Wilkinson and Pickett (2009) [7] noted greater economic equality generates wider social cohesion, lower crimes and more trust amongst citizens. All these have an impact on mental health.

The links between socio-economic factors and mental health play out in a number of complex ways. Wilkinson and Pickett (2017) [8] highlighted that psychosocial factors including the experience of ongoing acute stress can have detrimental impact on an individual’s mental health. The stigma attached to experiencing mental distress, despite public awareness raising campaigns, remains a factor in the development of health inequalities (Hatzebuehler et al. 2013) [9]. World Health Organisation (2014) [10] emphasised the social determinants of mental health. Silva et al. (2016) [11] outlined the potential impact of a range of socioeconomic factors on mental health. These include income inequality, poor housing and living in communities with a lack of resources. Research indicates that lower socioeconomic is a potential factor in suicidal behaviour (Platt et al. 2017) [12]. People from marginalised groups, for example asylum seekers and refugees or those who have experienced other forms of trauma, are more vulnerable to the development of mental health problems (Rafferty et al. 2015) [13]. The stresses of the daily experiences and pressures of living in poverty such as debt, worry about being able to cope with emergencies and precarious accommodation can all contribute to poor mental health Elliott (2016) [14]. Topor and Ljungqvist’s (2017) [15] research with service users demonstrates the positive impact of relatively small increases income can have. The increased income “*impacted their sense of self through experiences of mastery, agency, reciprocity, recognition and security*”.

Eaton’s (1980) [16] “*social drift*” hypothesis suggests that the onset of severe mental illness and its potential social consequences such as loss of employment and hospitalisation led to lower socioeconomic status. Health inequalities approaches argue that the roots of many of the issues that we now classify as mental illness fundamentally have their roots in poverty, social inequality and injustice (Marmot 2010; Karban 2016) [6,17]. The impact of austerity has been to deepen these inequalities. The United Nations (2016) [18] report, which was damning of the impact of austerity, specifically mentioned the poor provision of mental health care, alongside related issues such as the increased use of food banks and the rise in homelessness. The conclusion of the report was the policy of austerity had had a disproportionate impact on the most vulnerable. This is an inevitable conclusion that includes such drastic retrenchment. It is a statement of the obvious but one that bears repeating—welfare services, which were targeted, are designed to support those facing the most difficult situations. Any reduction in services will have its greatest impact on the poor and vulnerable. This is not by chance; it is a deliberate government policy—a war on the poor. The impact in the area of mental health is twofold. Firstly, such policies have a disproportionate impact on those with mental health problems. Secondly, such policies are bound to lead to higher levels of depression and anxiety amongst those are subjected to them. There are future costs as these policies increase the numbers of children living in poverty. There is a great deal of evidence that shows that growing up or living in poverty has long term implications for the onset of adult mental health problems.

## 2. Austerity

In 2008, the banking crisis led to the Labour Government of the UK, with Gordon Brown as Prime Minister, spending huge sums of money to bail out financial institutions. The banks were seen as “*too big to fail*”. As well as bailing out the banks, the Brown administration followed standard Keynesian economics by attempting to stimulate demand in the economy. These measures included a reduction in VAT and increased capital spending. The formation of the Coalition Government in 2010 ended this approach. The Coalition presented itself as a government formed in response to a national emergency, although it should be noted not representative of all major parties as is usually the case in such circumstances. The emergency was the position of the Government finances but the cause of that position was presented as the previous Labour government’s profligacy. Brown (2015) [19] noted that calls to individual sacrifice are an integral part of the discourse of the fiscal crisis as national emergency. In the UK context, the Tory Chancellor of Exchequer Osborne famous phrase that “*we are all in this together*” captures this.

Austerity was presented as a response to a national emergency. It was, as Krugman (2015) [20] concluded, a deeply political project. The aim was to reduce the extent and range of welfare provision across a number of areas. This reduction was not to be temporary—it would be a permanent recasting of the role of welfare and the relationship between the individual and the state. The welfare state does not simply exist to offer protections to the most vulnerable (Hills 2017) [21]. However, in the period of austerity—that is the retrenchment of services—it is those who are in most of need of services who suffer most. This group includes people with severe mental health problems who are in receipt of out of work benefits.

In the UK, the impact of the 2008 banking crisis continues to be felt across the public and welfare sectors. The bail out of the banks—an act of government intervention to rescue the loudest supporters of the free market—required the injection of huge sums of public money. Oxfam (2013) [22] estimated that the cost was £141 billion. Austerity as a policy involves huge cuts in public and welfare spending. Krugman (2015) [20] noted that austerity marks a significant departure from traditional management of the economy as a fiscal stimulus would normally be introduced in an economic downturn.

Austerity cannot be understood as purely or solely a matter of economics. It was a clear political project to recast and reduce the role of the social state (Cummins 2018) [23]. The Coalition Government successfully put forward the message that the crisis in the public finances was the result of the previous administration’s failure to control public spending during a period when the economy was experiencing sustained growth. The measures that were introduced were ones that would reduce welfare provision but also shrink the social state permanently. Blyth (2013) [24] was highly critical of austerity on economic terms but also the morality of the poor being made to pay for a situation created by the one of the wealthiest groups in society. As Taylor-Gooby (2012) [25] concluded, the result of austerity is that the UK public sector has shrunk to the lowest amongst major economies. This includes the United States. Taylor-Gooby (2012) [25] concluded that the attack on the welfare state was the most sustained that it had faced. Cameron’s (2015) [26] notion of a leaner more business-like “*smarter state*” involves a recasting of the relationship between individuals, communities and the state.

A Joseph Rowntree Foundation (JRF 2016) [27] report shows that the majority of those living in poverty are actually in work. The extension of zero-hour contracts, the 1% cap on public sector pay and the cuts in services combine to increase inequality but also the sense of precariousness in employment. The Institute for Fiscal Studies (IFS 2012) [28] has calculated that there will be a reduction of over 900,000 public sector jobs in the period 2011–2018. The public sector as well as doing vitally important work for the wider society is also an area that employs more women than other sectors of the economy. Therefore, the impact of austerity has to be viewed through the prism of gender alongside race, class and disability. The current Tory Government under Prime Minister Mrs May has stopped using the term austerity and is attempting to distance itself from the policies of the UK Coalition Government of 2010–2015. However, there are more cuts to public services planned alongside welfare reforms that will impact disproportionately on the poorest, particularly families with children (Crossley 2016) [29]. Far from being “all in it together”, it is clear that the most vulnerable have paid the greatest price. Beatty and Fothergill (2016) [30] calculated that their will be reductions in welfare spending of £27 billion a year by 2020–2021. The Coalition’s commitment to localism and the withdrawal of funding linked to deprivation means that the local authorities with the highest levels of need have had to manage the most significant reductions (Innes and Tetlow 2015) [31]. This is Tudor-Hart’s (1971) [32] inverse care law as government policy with those in most need being allocated fewest resources.

Crossley (2016) [29] concluded that the largest cuts were experienced in those areas that had officially been identified as being poorer and having increased local needs. There is a vicious circle here as individuals and families in these areas are less likely to have the economic and social capital to replace the resources and community assets that are not sustainable without state funding. Bourdieu et al. (1999) [33] concluded that the poorest areas of our cities become characterised by *“absence”*—i.e., they have been largely abandoned by the welfare institutions of the state.

Austerity has had a profound impact on the welfare state and those who are most reliant on the services it provides. The Coalition government’s policies, therefore inevitably had the most impact on the most vulnerable. Fifty per cent of the cuts in spending fell in two areas: benefits and local government spending (Centre for Welfare Reform, 2015) [34]. These areas account for twenty-five per cent of government spending. Austerity led to a 20% cut in benefits—the majority of which are paid to people with disabilities and people living in poverty. As outlined above, the cuts in local government spending have disproportionately impacted on the poorest and the poorest communities. People living with severe disabilities bear 15% of the cuts in welfare spending. Welfare in the debates around austerity was a very specific term and was always linked with dependency (Garrett 2015) [35]. It meant payments to those not in work. Pensions were seen as having a different status—also the recipients were seen as an important political constituency. The Government’s so-called “triple lock” on pensions meant that they were unaffected. The triple lock guarantees that the basic state pension will rise by a minimum of 2.5%, the rate of inflation or average earnings growth, whichever is largest. Before 2011, the state pension rose in line with the retail prices index (RPI) measure of inflation. This has been consistently lower than annual rises in earnings or 2.5%.

## 3. The Impact of Welfare Reforms

One of the key ideas in the neoliberal attack on the welfare state is that it creates dependency. In addition, it is felt that the payment of benefits allows the claimant to avoid responsibility. It has long been the aim of these critics of the welfare state to place more conditions on benefits—the reforms to the welfare system introduced by the Coalition Government building on initial moves by the previous Labour Government, led by Prime Minister Gordon Brown, did this. In particular, the Work Capability Assessment (WCA) scheme meant that individuals who were claiming the Employment and Support Allowance (ESA) underwent a fitness to work assessment. ESA is a benefit that is paid to individuals with an illness, health condition or disability that restricts or makes it impossible for them to work. The WCA was introduced in 2008. Originally, the scheme was managed by a private IT company ATOS who won the contract from the Department of Work and Pensions (DWP), the government department responsible for these issues. The WCA is still outsourced to Maximus, a private organisation, that carries out the assessment for the DWP. This is an example of the processes whereby state functions are privatised and then managed for profit by commercial companies. It also highlights the way that the state contracts have become key markets for private sector companies.

There are clear barriers to employment that people living with physical disabilities or mental health problems face. Access to employment with good pay and conditions is clearly an aim that people would support. The WCA scheme was presented by the DWP as an attempt to help people off benefits and into employment. This is a laudable aim but the experience of the scheme was somewhat different. It was an essentially punitive experience. In the assessment process, there are three possible outcomes:

fit for work

unfit for work but fit for pre-employment training

fit for neither work nor training

One of the most significant criticisms of the WCA is that the assessment is a functional one. It is often carried out by staff who have little understanding or professional experience of mental health problems. By their nature, mental health problems are complex, fluctuating and therefore difficult to assess. The impact on an individual’s ability to work may vary across various forms of employment. It will also change because of the nature of the difficulties that they face. For example, low mood or anxiety are symptoms of illness that would be difficult to evidence in the way that bureaucracies demand. All these factors combine to put people with mental health problems at a disadvantage if a testing regime is a functional one—focusing on physical capabilities. There have been concerns not only about the outcome of the WCA—in terms of people being deemed fit for work whose mental health was still such that they were not—but also the impact of the whole WCA process.

An analysis of the introduction of the WCA process by Barr et al. (2016) [36] provides a clear insight into the impact of this new system. The study examines the programme in the period 2010–2013. There were just over a million assessments in this period. The number of assessments varied across the country. The study concluded that in those areas with higher rates of reassessment, there was an increase in suicides, anti-depressant prescribing and self-reported mental health problems. The authors concluded that, across England, the WCA process was linked to:

590 suicides

279,000 additional cases of self-reported mental health problems

725,000 additional prescriptions for anti-depressants

Those in the lowest socio-economic groups are more likely to be in receipt of these benefits. The social gradient in health (Marmot 2010) [2] means there is a greater prevalence of mental and physical health problems within this cohort. The WCA process thus has a double impact, as it is more likely to affect vulnerable individuals in more marginalised communities. In addition, this research focused on those who were subject to the WCA process. The wider social impacts also need to be examined. The 590 people who ended their own lives were all members of families, communities and wider social networks. The impacts of such policies are even broader than is sometimes allowed for.

Barr et al. (2016) [36] regarded the whole WCA process as an experiment in public health that had disastrous consequences for individuals, families and communities. The authors concluded that any benefits in terms of reduced welfare spending are far outweighed by the personal and wider damage that the policy caused. It may have actually lead to increased public spending in terms of greater calls on mental health and other agencies. Such policies do not tackle the exclusion of people with mental health problems from the labour market. It might well be that they increase rather than decrease this exclusion.

Stuckler and Basu (2013) [37] showed that the shredding of the social state has clear negative impacts on health outcomes. The example of Greece which saw the welfare and health budget slashed as part of the Eurozone bailout is a very pertinent example. In April 2012, Greece was shocked by the suicide of a 77-year-old pensioner, a retired pharmacist who shot himself outside the parliament building in Athens. In a note, he stated that he had decided to end his life as he did not want to be reduced to foraging in bins for food. The suicide encapsulated the despair of many of the older generation in Greece. In this period, pensions and other benefits were cut by up to 25% as part of the conditions of the loans that the government received to tackle the sovereign debt crisis. Prior to the economic crisis, Greece had one of the lowest suicide rates in the EU. Antonakakis et al. (2016) [38] explored the relationship between austerity and suicide. The paper examines trends across five countries in the “*Eurozone periphery*”: Greece, Italy, Ireland, Portugal and Spain. Fiscal austerity has had an impact on the suicide rate in these countries. The authors concluded that these policies of retrenchment have short-, medium- and long-term impacts on the suicide rates of males. A 1% reduction in government spending is associated with increases of 1.38%, 2.42% and 3.32% in the short, medium and long-term male suicide rates, respectively.

There is a danger that, even though attitudes have shifted to incorporate wider perspectives on the social factors that cause mental distress, the responses or solutions are viewed in terms of very traditional service models. This is one of the criticisms of radical groups such as Recovery in the Bin [39]. This perspective is an overtly political one linking mental health issues to neoliberal political ideas in a very explicit way. It is not simply that austerity has led to cuts in services, it is argued that the neoliberal economy generates a range of mental health problems. This is a modern recasting of the arguments of the anti-psychiatry movement (Cummins 2017) [40]. Thinkers such Laing and Foucault used their criticisms of psychiatry as a base, from which, to mount a broader act on capitalism. There are a number of examples of community and other groups that tackle the impact of mental health problems. It is in these dynamic community based, service user led models that the progressive values that led to deinstitutionalisation still flourish. Black, Asian, and Minority Ethnic (BAME) is a term used to refer to members of non-white communities in the UK. Many local and national BAME groups such as Black Mental Health UK [41] continue to challenge institutional racism and the overrepresentation of people from BAME backgrounds in mental health services.

Service user and campaigning groups have had a significant impact on mental health services. However, there is a concern that this can lead to tokenistic responses from service providers and mental health professionals. Recovery in the bin name sums this up. The group rejects what it sees as the “*colonisation*” of the term recovery. In effect, it has become a management buzz word or phrase drained of its radical and critical perspectives. Durand and co-workers’ (2014) [42] review of shared decision making in health services has to be seen in the wider context of health inequalities. It is not specifically concerned with mental health services but there are wider lessons to be drawn. I would argue that some of these, about the need to develop approaches that foster the genuine involvement of patients in decisions about their care, are particularly relevant to mental health services.

One of the frustrations of modern health and social care service provision is the consistent parroting of phrases such as “*No decision about me, without me*” which do not then result in real change in the organisation and delivery of services. “Shared Decision Making” (SDM), which is now widely considered one of the goals of healthcare services to encourage patients to take greater responsibility for their own health, has two elements: lifestyle choices and private insurance. In addition, policy makers seek to ensure that patients are more heavily involved in decisions about their treatment. The positives of this approach are that patients will increase their knowledge and therefore be in a position to make more informed choices. The impact of welfare reforms has seen professional groups allying with service users to highlights the impact and injustice of these measures. For example, Psychologists Against Austerity is a group that campaigns against welfare reform:

“*As applied psychologists in the UK we believe it is our public and professional duty to be speaking out against the further implementation of austerity policies. From professional experience and our knowledge of empirical psychological evidence, we know that cuts have been toxic for people’s wellbeing and mental health.*”

[43].

The impacts of major mental illness are clearly social as they affect people’s opportunities to work and as discussed here establish social networks. There is a loop here as this social marginalisation, by its nature, has potentially damaging impacts on individual’s mental health and sense of well-being. Social work as a profession and discipline, alongside the others in mental health services should have social inclusion and social justice at its core. Newlin and co-workers’ (2015) [44] systematic review of fourteen social network approaches identified four broad domains.

*Asset based approaches*: These approaches focused on developing the capacities of individuals and communities. This group included, for example, art therapy or similar activities. The importance of such activities lies not just in the activity itself but the mutual support and safety that a group can provide. These approaches help to improve self-esteem and confidence.

*Social Skills development*: Three of the group interventions focused on social skills development. The most commonly taught skills included social problem solving, life skills, listening and communication and activity planning. They have to be viewed in the broadest social and cultural terms.

*Goal setting:* This approach was often based on a case management model. The relationship between the participants and workers was identified as the main driver in the improvement in social networks.

*Peer support*: The broader advantages of peer support are well documented. The small nature of the sample makes it difficult to reach any firm conclusions.

Such approaches are based in fundamental values of mutuality and respect but also require mental health professionals to shifting their thinking and approach. In addition, there are several examples of agencies that one might not expect respond to mental health issues. I will use the example of Network Rail’s response to suicide on the railways. Network Rail, the organisation that has overall responsibility for managing the railway system, is a signatory of the Mental Health Crisis Care Concordat (2104) [45]. The Concordat was signed by 22 national bodies. It acknowledged that there was a need for a new approach from services in their response to those in acute mental distress. Network Rail has a pivotal role working with other organisations such as the British Transport Police and Samaritans to reduce the number of suicides on the railway. Samaritans organise to visit after an incident to offer support to staff and passengers. It has been involved in training over 11,000 rail staff on how to identify, approach and support a potentially suicidal person. Network Rail in partnership with the British Transport Police and Samaritans have identified priority locations. A range of responses are being developed. These include physical changes, such as more fencing, barriers at the end of platforms and alterations to the design of stations.

## 4. Austerity and Mental Health Services in the UK

Beresford (2013) [46] produced a devastating critique of the position of mental health services within the UK. He noted that there was a seemingly ever widening gap between a series of Government mental health policy documents, which consistently promised the completion of a mental health service revolution and the reality of service provision. For all the talk of a complete shift in focus in the structure and values in services, a series of issues remain to be addressed. These include the use of compulsory powers under the Mental Health Act (MHA 1983), access to services for those in crisis, the involvement of the police and other criminal justice agencies in mental health and the ongoing need for the meaningful involvement of service users in the development and delivery of mental health services.

A report from London School of Economics (LSE 2012) [47] concluded that, within the UK, mental illness now constitutes nearly half of all ill health suffered by people under 65—yet only a quarter of those involved are in any form of treatment. Mental illness accounts for 23% of the total burden of disease. However, despite the existence of cost-effective treatments, it receives only 13% of National Health Service (NHS) health expenditure. The LSE report highlighted the following:

*Pressures on services made them increasingly difficult to access. People in mental health crisis face real barriers to access appropriate support.*

*BAME service users were more likely to be subject to the MHA and at the same time were less likely to have access to talking therapies or other alternatives to medication.*

*People with learning difficulties and older people continue to be poorly served by both mental health and physical care services.*

*The damaging impact of welfare and benefit reforms had a direct impact on the mental health of individuals.*

In one of her first speeches as Prime Minister, Theresa May highlighted the current difficulties in mental health services as one of the “*burning injustices*” that her premiership would set out to tackle. In announcing the Wessley review of the Mental Health Act, Mrs May highlighted the overrepresentation of people from Black and Minority Ethnic (BAME) communities in the number of patients detained against their will using formal powers There is a danger of concentrating on the legal processes to the detriment of asking broader questions about psychiatry—and mental health services’ reliance on coercion. The well documented rise in the number of detentions under the MHA in England and Wales can be seen as one indicator or symptom of a more fundamental issue—the lack of community based services that can provide appropriate support to those in crisis.

## 5. Conclusions

The damage that nearly a decade of austerity has done to mental health services is apparent in a number of areas including—the pressure on inpatient beds, out of area placement, the response to individuals in crisis, the increased use of the MHA, the position of the CJS as a default provider of mental health care and the personal toll that policies such as the WCA have inflicted. Services faced increased pressures to do more with less. Any visitor to a major city in England must be immediately shocked by the increasing numbers of homeless people living on the streets. The “poverty aware paradigm” that Krumer-Nevo (2015) [48] outlined can be adapted or applied within the mental health settings by professionals from all backgrounds. This involves “facing both ways”—i.e., working with individuals and within communities. The poverty aware paradigm starts from the proposition that poverty is a fundamental violation of individual human rights. It can also be viewed as the denial of human rights to groups of citizens. Kelly (2005) [49] used the term “structural violence” to describe the combined impact of factors such poverty, race and mental health status on individuals and communities. Bourdieu (2010) [50] argued that language is not only a means of communication but is also a medium of power. User/survivor may not be a term that is widely used by psychiatrists, doctors will, on the whole, use the term patients. This is, of course, a reflection of what they see as the power dynamics of these relationships. The term user/survivor did have important ramifications and influence in the broader mental health field. In health generally, there has been a shift towards a more consumerist approach. This is partly a reflection of wider cultural trends. However, as Karban (2016) [17] noted, this also has the potential to create a narrative of individual responsibility that hides the structural causes of health inequalities. The consumer movement in health helped to create a space for the survivor in mental health. Crossley (2004, p. 167) [51] argued that:

“*survivors have been able to convert their experiences of mental distress and (mis)treatment into a form of cultural and symbolic capital. The devalued status of the patient is reversed within the movement context.*”

There are new areas or approaches such as Trauma Informed Approaches (Sweeney et al. 2016) [52] and the building of social capital (Webber et al. 2015) [53] that have potential to make further progress in attitudes and responses to mental distress. Such approaches focus on the lived experiences of those who use mental health services. These perspectives recognise that institutionalised responses have the potential to inflict more harm. In addition, they acknowledge that even now the response to mental distress remains heavily focused on medical interventions. This paradigm shift can only be successful in the context of the development of a broader range of policies that are committed to reducing social inequality and tackling social injustice. This is the core of social work values and practice. The optimism of the initial period of deinstitutionalisation was replaced, during the 1980s and 1990s, by wider societal concerns that focused on risk. As services have come under increasing pressures, systems have developed that focus on these issues and marginalise user/survivor voices. There is an alternative which is to see the current position as an opportunity or an opening to create a new debate about the causes of and societal responses to mental distress. Within these debates, a consideration of the structure and role of mental health services should be a key element. This would involve a shift in focus so that mental health and broader welfare services address structural issues rather than focusing on individualised models of mental distress.

## References

[1] Scull A. (2015). Madness in Civilization.

[2] Foot J. (2015). The Man Who Closed the Asylums: Franco Basaglia and the Revolution in Mental Health Care.

[3] Macintyre A., Ferris D., Gonçalves B., Quinn N. (2018). What has economics got to do with it? The impact of socioeconomic factors on mental health and the case for collective action. Palgrave Commun..

[4] Shim R., Koplan C., Langheim F.J.P., Manseau M.W., Powers R.A., Compton M.T. (2014). The social determinants of mental health: An overview and call to action. Psychiatr. Ann..

[5] Murray R. (2017). Mistakes I have made in my research career. Schizophr. Bull..

[6] Wilkinson R., Pickett K. (2009). The Spirit Level: Why Equality is Better for Everyone.

[7] Marmot M. (2010). Fair Society, Healthy Lives, The Marmot Review.

[8] Wilkinson R., Pickett K. (2017). The enemy between us: The psychological and social costs of inequality. Eur. J. Soc. Psychol..

[9] Hatzenbuehler M., Phelan J., Link B. (2013). Stigma as a fundamental cause of population health inequalities. Am. J. Public Health.

[10] World Health Organization and Calouste Gulbenkian Foundation (WHOCG) (2014). Social Determinants of Mental Health.

[11] Silva M., Loureiro A., Cardoso G. (2016). Social determinants of mental health: A review of the evidence. Eur. J. Psychiatry.

[12] Platt S., Stace S., Morrissey J. (2017). Dying From Inequality: Socioeconomic Distadvantage and Suicidal Behaviour.

[13] Rafferty J.A., Abelson J.M., Bryant K., Jackson J.S., Compton M.T., Shim R.S. (2015). Discrimination. The Social Determinants of Mental Health.

[14] Elliott I. (2016). Poverty and Mental Health: A Review to Inform the Joseph Rowntree Foundation’s Anti-Poverty Strategy.

[15] Topor A., Ljungqvist I. (2017). Money, social relationships and the sense of self: The consequences of an improved financial situation for persons suffering from serious mental illness. Community Ment. Health J..

[16] Eaton W. (1980). A formal theory of selection for schizophrenia. Am. J. Sociol..

[17] Karban K. (2016). Developing a health inequalities approach for mental health social work. Br. J. Soc. Work.

[18] UN Committee on the Rights of Disabled Persons (CRPD) (2016). Inquiry Concerning the United Kingdom of Great Britain and Northern Ireland Carried out by the Committee Under Article 6 of the Optional Protocol to the Convention.

[19] Brown W. (2015). Undoing the Demos.

[20] Krugman P. (2015). The Case for Cuts Was a Lie. Why Does Britain Still Believe It?. The Guardian.

[21] Hills J. (2017). Good Times, Bad Times (revised Edition): The Welfare Myth of Them and Us.

[22] Oxfam (2013). Truth and Lies about Poverty: Ending Comfortable Myths about Poverty. www.oxfam.org.

[23] Cummins I. (2018). Poverty, Inequality and Social Work: The Impact of Neo-liberalism and Austerity Politics on Welfare Provision.

[24] Blyth M. (2013). Austerity: History of a Dangerous Idea.

[25] Taylor-Gooby P. (2012). Overview: Resisting welfare state restructuring in the UK. J. Poverty Soc. Justice.

[26] Cameron D. (2015). My Vision for a Smarter State. https://www.gov.uk/government/speeches/prime-minister-my-vision-for-a-smarter-state.

[27] Tinson A., Ayrton C., Barker K., Born T.B., Aldridge H., Kenway P. (2016). Monitoring Poverty and Social Exclusion 2016. Children.

[28] Institute for Fiscal Studies (2012). Reforming Council Tax Benefit, IFS Commentary 123. www.ifs.org.uk/comms/comm123.pdf.

[29] Crossley S. (2016). The Troubled Families Programme: In, for and against the state?. Soc. Policy Rev. Anal. Debate Soc. Policy.

[30] Beatty C., Fothergill S. (2016). The Uneven Impact of Welfare Reform: The Financial Losses to Places and People.

[31] Innes D., Tetlow G. (2015). Delivering fiscal squeeze by cutting local government spending. Fiscal Stud..

[32] Tudor Hart J. (1971). The inverse care law. Lancet.

[33] Bourdieu P., Ferguson P.P. (1999). The Weight of the World: Social Suffering in Contemporary Society: Social Suffering and Impoverishment in Contemporary Society.

[34] Centre for Welfare Reform (2015). A Fair Society.

[35] Garrett P.M. (2015). Words matter: Deconstructing “welfare dependency” in the UK. Crit. Radic. Soc. Work.

[36] Barr B., Taylor-Robinson D., Stuckler D., Loopstra R., Reeves A., Wickham S., Whitehead M. (2016). Fit-for-work or fit-for-unemployment? Does the reassessment of disability benefit claimants using a tougher work capability assessment help people into work?. J. Epidemiol. Community Health.

[37] Stuckler D., Basu S. (2013). The Body Economic: Why Austerity Kills.

[38] Antonakakis N., Collins A. (2016). The impact of fiscal austerity on suicide mortality: Evidence across the ‘Eurozone periphery’. Soc. Sci. Med..

[39] Recovery in the Bin. https://recoveryinthebin.org/.

[40] Cummins I. (2017). Critical Psychiatry: A Biography.

[41] BMH UK http://www.blackmentalhealth.org.uk/.

[42] Durand M.-A., Carpenter L., Dolan H., Bravo P., Mann M., Bunn F., Elwyn G. (2014). Do Interventions Designed to Support Shared Decision-Making Reduce Health Inequalities? A Systematic Review and Meta-Analysis. PLoS ONE.

[43] The Psychological Impact of Austerity. http://www.psychchange.org/psychologists-against-austerity.html.

[44] Newlin M., Webber M., Morris D., Howarth S. (2015). Social Participation Interventions for Adults with Mental Health Problems: A Review and Narrative Synthesis. Soc. Work Res..

[45] Mental Health Crisis Care Concordat. https://www.crisiscareconcordat.org.uk/.

[46] Beresford P. (2013). Mental Health is in No Fit State, Whatever the Politicians Say. https://theconversation.com/mental-health-is-in-no-fit-state-whatever-the-politicians-say-15743.

[47] LSE (2012). How Mental Illness Loses Out in the NHS. http://cep.lse.ac.uk/pubs/download/special/cepsp26.pdf.

[48] Krumer-Nevo M. (2015). Poverty-aware social work: A paradigm for social work practice with people in poverty. Br. J. Soc. Work.

[49] Kelly B. (2005). Structural violence and schizophrenia. Soc. Sci. Med..

[50] Bourdieu P. (2010). Distinction.

[51] Crossley N. (2004). Not being mentally ill: Social movements, system survivors and the oppositional habitus. Anthropol. Med..

[52] Sweeney A., Clement S., Filson B., Kennedy A. (2016). Trauma-informed mental healthcare in the UK: What is it and how can we further its development?. Ment. Health Rev. J..

[53] Webber M., Reidy H., Ansari D., Stevens M., Morris D. (2015). Enhancing social networks: A qualitative study of health and social care practice in UK mental health services. Health Soc. Care Community.

